# Pulmonary Macrophages Attenuate Hypoxic Pulmonary Vasoconstriction via β_3_AR/iNOS Pathway in Rats Exposed to Chronic Intermittent Hypoxia

**DOI:** 10.1371/journal.pone.0131923

**Published:** 2015-07-01

**Authors:** Hisashi Nagai, Ichiro Kuwahira, Daryl O. Schwenke, Hirotsugu Tsuchimochi, Akina Nara, Sayoko Ogura, Takashi Sonobe, Tadakatsu Inagaki, Yutaka Fujii, Rutsuko Yamaguchi, Lisa Wingenfeld, Keiji Umetani, Tatsuo Shimosawa, Ken-ichi Yoshida, Koichi Uemura, James T. Pearson, Mikiyasu Shirai

**Affiliations:** 1 Department of Forensic Medicine, Tokyo Medical and Dental University, Tokyo, Japan; 2 Department of Forensic Medicine, The University of Tokyo, Tokyo, Japan; 3 Department of Clinical Laboratory Medicine, The University of Tokyo, Tokyo, Japan; 4 Department of Pulmonary Medicine, Tokai University Tokyo Hospital, Tokyo, Japan; 5 Department of Physiology, Heart Otago, University of Otago, Dunedin, New Zealand; 6 Department of Cardiac Physiology, National Cerebral & Cardiovascular Center, Osaka, Japan; 7 Department of Pathology and Microbiology, Nihon University School of Medicine, Tokyo, Japan; 8 Institute of Forensic Medicine, Ludwig-Maximilians-University Munich, Munich, Germany; 9 Japan Synchrotron Radiation Research Institute, Hyogo, Japan; 10 Department of Forensic Medicine, Tokyo Medical University, Tokyo, Japan; 11 Monash Biomedical Imaging Facility and Department of Physiology, Monash University, Melbourne, Australia; 12 Imaging and Medical therapy Beamline, Australian Synchrotron, Clayton, Australia; University of Giessen Lung Center, GERMANY

## Abstract

Chronic intermittent hypoxia (IH) induces activation of the sympathoadrenal system, which plays a pivotal role in attenuating hypoxic pulmonary vasoconstriction (HPV) via central β_1_-adrenergic receptors (AR) (brain) and peripheral β_2_AR (pulmonary arteries). Prolonged hypercatecholemia has been shown to upregulate β_3_AR. However, the relationship between IH and β_3_AR in the modification of HPV is unknown. It has been observed that chronic stimulation of β_3_AR upregulates inducible nitric oxide synthase (iNOS) in cardiomyocytes and that IH exposure causes expression of iNOS in RAW264.7 macrophages. iNOS has been shown to have the ability to dilate pulmonary vessels. Hence, we hypothesized that chronic IH activates β_3_AR/iNOS signaling in pulmonary macrophages, leading to the promotion of NO secretion and attenuated HPV. Sprague-Dawley rats were exposed to IH (3-min periods of 4–21% O_2_) for 8 h/d for 6 weeks. The urinary catecholamine concentrations of IH rats were high compared with those of controls, indicating activation of the sympathoadrenal system following chronic IH. Interestingly, chronic IH induced the migration of circulating monocytes into the lungs and the predominant increase in the number of pro-inflammatory pulmonary macrophages. In these macrophages, both β_3_AR and iNOS were upregulated and stimulation of the β_3_AR/iNOS pathway *in vitro* caused them to promote NO secretion. Furthermore, *in vivo* synchrotron radiation microangiography showed that HPV was significantly attenuated in IH rats and the attenuated HPV was fully restored by blockade of β_3_AR/iNOS pathway or depletion of pulmonary macrophages. These results suggest that circulating monocyte-derived pulmonary macrophages attenuate HPV via activation of β_3_AR/iNOS signaling in chronic IH.

## Introduction

Intermittent hypoxia (IH) during sleep periods is a distinctive feature in the patients of sleep apnea syndrome (SAS) [1–3]. IH exposure to healthy humans and animals causes prolonged activation of the sympathoadrenal system and elevation of daytime blood pressure [1, 4–8]. Therefore, IH accompanying sympathoadrenal activation has been implicated in the pathogenesis of systemic hypertension caused by SAS [9]. However, the effect of an increase in sympathoadrenergic activity on pulmonary vascular tone is not fully elucidated.

Hypoxic pulmonary vasoconstriction (HPV) is an important mechanism for optimizing ventilation/perfusion matching [10] and also inducing pulmonary hypertension [2]. In a previous study, we have reported that the centrally-mediated increase in sympathetic nervous activity following IH acts to blunt HPV via β_1_-adrenergic receptors (β_1_AR) in the brain [11]. In addition, we have also reported that IH-derived activation of β_2_AR, not β_1_AR, in the pulmonary arteries attenuates the HPV [12]. These results demonstrate that IH-derived sympathoadrenal activation attenuates HPV via β_1_AR and β_2_AR. However, the role of β_3_AR in modifying HPV is unknown.

In *in vivo* and *in vitro* animal experiments, it has been demonstrated that diseases associated with prolonged increase in catecholamine levels result in β_3_AR upregulation in cardiomyocytes [13–16]. Furthermore, chronic stimulation of β_3_AR has been shown to induce inducible nitric oxide synthase (iNOS) overexpression and NO secretion in the mouse heart [17]. It is interesting to note that IH exposure per se increased iNOS expression in RAW264.7 macrophages *in vitro* [18]. Moreover, iNOS/NO signaling has the ability to dilate pulmonary vessels during septic shock [19, 20]. These studies suggest a possibility that iNOS in pulmonary macrophages is upregulated by chronic IH directly and/or chronic stimulation of β_3_AR in response to the IH-induced sympathoadrenal activation to release NO and reduce pulmonary vascular tone. To the best of our knowledge, however, there has been no report concerning activation of β_3_AR and iNOS/NO signaling in pulmonary macrophages following chronic IH.

In this study, we hypothesized that 1) β_3_AR in the pulmonary macrophages may be upregulated by IH associated with sympathoadrenal activation, 2) iNOS expression in the pulmonary macrophages may also be increased following stimulation of the upregulated β_3_AR as well as directly by IH per se, and 3) the β_3_AR/iNOS signaling in the pulmonary macrophages may be activated to release substantial NO in response to acute hypoxic exposure and thus, may modify HPV. To verify these hypotheses, we performed the following experiments using rats treated with chronic IH (IH rats) and rats exposed to normoxia (N rats). We first evaluated the expression of β_3_AR and iNOS in the pulmonary macrophages using immunohistochemical and electrochemical techniques (i.e. Western blot). Second, we examined whether stimulation of β_3_AR on the macrophages promotes iNOS-mediated NO secretion *in vitro* using bronchioalveolar lavage (BALF)-derived macrophages. Third, using synchrotron radiation microangiography for visualizing the pulmonary microvessels *in vivo*, we revealed the functional contribution of β_3_AR/iNOS signaling in the pulmonary macrophages to modulation of HPV.

## Materials and Methods

### Animals

Experiments were conducted on 7 wk old male Sprague-Dawley rats. All rats were on a 12: 12-h light-dark cycle at 25°C and were provided with food and water ad libitum. Rats were divided into two groups. One was housed in normoxic conditions (N rats). The other was continuously housed in an airtight Plexiglas chamber (27 x 44 x 19 cm, model KYN-370, Bioresearch Center, Tokyo, Japan) with IH exposure for 6 weeks, except for a 10 minute interval every fifth day when chamber was cleaned (IH rats) [21]. IH treatment consisted of alternating 90 seconds cycles of normoxia (21% O_2_) and hypoxia (reaching 4% O_2_ at the nadir). N_2_ was delivered to the chamber at a rate of 14 L/min (PSA type N_2_ generator, ECONOX Ver. 2.10, ECOTS, Osaka, Japan). Compressed air was delivered at a rate of 54 L/min (Oil free scroll type, Smart Air SLP-15EBD, ANEST IWATA, Yokohama, Japan). The gas flushing the chamber was automatically switched from compressed air to N_2_ and back to compressed air with the use of a timed solenoid valve. The O_2_ concentration in the chamber was monitored by an O_2_ analyzer (Oxygen monitor JKO-25 Ver. 3, JIKCO, Tokyo, Japan). Exposure was performed 8 hours/day (9:00 AM—5:00 PM) for 42 consecutive days. All experiments were approved by the Institutional Animal Care and Use Committee of the University of Tokyo.

### Sampling of 24-hour Urine Catecholamine

For 24-hour urine collection, N rats and IH rats were housed in metabolic cages (Natsume, Tokyo, Japan) at the completion of the 6-week IH exposure. The collected urines were consecutively drawn into tubes containing 20 μL of 2.5 mol/L HCl. After urine collection, concentration of catecholamine was measured using high-performance liquid chromatography method (SRL Japan, Tokyo, Japan).

### Intratracheal Administration of Liposomal Clodronate

Liposomal clodronate was administered into the trachea as previously described [22]. It was reported previously that the most effective depletion of macrophage was observed after 72 h of clodronate intratracheal administration (i.t.) [22, 23]. After 6 weeks exposure of IH, a single dose of liposomal clodronate (Clodrosome, Encapsula NanoSciences, Nashville, TN) was administered to N and IH rats via the i.t. route 3 days before synchrotron radiation (SR) microangiography. Rats were sedated with pentobarbital (70 mg/kg) intraperitoneally (i.p.). Using standard aseptic procedures, the tracheas were exposed by surgical resection and pierced with a 26-gauge needle for i.t. injection of 500 μg of clodronate contained in 100 μL saline. The neck wound was closed with sterile sutures. To confirm that the i.t. injection of clodronate depletes pulmonary macrophages, the lungs were isolated immediately after clodronate treatment in another group of N and IH rats. In this case, the reduction in number of macrophages was evaluated with immunofluorescent staining using anti-ED-1 antibody (see below).

### Chronic Repeated Intravenous Administration of Fluorescent Liposomes

Fluorescent liposomes (Fluoroliposome, Encapsula NanoSciences) were injected via the caudal vein on -1 day before IH exposure, and injection was performed every week during the 6 weeks of IH experiment. Each time 0.4 mL of fluorescent liposome contained in 1 mL saline was injected. Lung and liver tissues were frozen in O.C.T., and sliced into 10-μm sections with a cryostat. Images of the unfixed cryosections were captured with fluorescence microscopy BIOREVO BZ-9000 (Keyence, Osaka, Japan). The liver sections were used as a positive control for circulating monocytes-derived macrophages which engulf fluorescent liposomes.

### Synchrotron Radiation Microangiography

SR pulmonary microangiography was performed as described previously [24]. Each rat was anesthetized with pentobarbital sodium (70 mg/kg, i.p.) and analgesic agent butorphanol tartrate (0.5 mg/kg, i.p.). Supplementary doses of pentobarbital (~15 mg/kg/hr i.p.) and butorphanol tartrate (0.025 mg/kg/hr i.p.) were periodically administered to maintain a surgical level of anesthesia during microangiography procedure. We used 5-min exposure to 10% O_2_ to induce HPV. In this study, 4 types of protocol were performed. 1) To assess the β_3_AR mediated modification of HPV, an angiogram during hypoxic exposure was recorded following the baseline angiogram with room air. These angiograms were repeated after administration of SR59230A (lipophilic selective β_3_-blocker, 7.5 mg/kg, i.v., Sigma-Aldrich) in N and IH rats. 2) To assess β_3_AR/NOS signaling-mediated pulmonary vasodilation, the angiograms were recorded before and after acute administration of CL316243 (lipophilic selective β_3_-agonist, 100 μg/kg, i.v., Tocris Bioscience, Ellisville, MO, USA) with/without L-NAME (non-selective NOS blocker, 50 mg/kg, i.v., Sigma-Aldrich) or L-NIL (selective iNOS blocker, 3 mg/kg, i.v., Cayman Chemical, Ann Arbor, MI). All angiograms in this protocol were taken under ganglion blockade with hexamethonium bromide (autonomic ganglionic blocker, 25 mg/kg, i.v., Wako, Osaka, Japan) to exclude the secondary effect of the β_3_-agonist via central nervous system. 3) To assess the modification of HPV by iNOS, the angiogram during acute hypoxic exposure was recorded following the baseline angiogram with room air, and these angiograms were repeated after an administration of L-NIL in N and IH rats. 4) To assess the modification of HPV by IH-derived accumulated pulmonary macrophage, the angiograms with room air and hypoxic exposure were recorded in N and IH rats following i.t. administration of liposomal clodronate 3 days before angiography.

### Image Analysis of SR Microangiograms

Image analysis was performed using Image Pro-Plus ver. 4.1 (Media Cybernetics, Silver Spring, MD) as described previously [24]. The line-profile function of Image Pro-Plus was used as an accurate method for measuring the internal diameter (ID) of individual vessels. A 50 μm thick tungsten wire appeared in all recorded images and was used as a reference for calculating vessel ID. Vessels were categorized according to ID; 100–200 μm, 200–300 μm, 300–500 μm and 500- μm. The magnitude of HPV was calculated as % change in diameter relative to baseline.

### Immunohistochemistry of Lung Sections

Paraffin blocked lungs from N and IH rats were sliced into 5-μm sections and placed onto clean glass slides. After deparaffinization, the slides soaked in the citrate buffer were heated with microwave for 5 min for antigen retrieval. Then, the slides were incubated in methanol with 3% hydrogen peroxide for 10 min to block endogenous peroxidase activity. Nonspecific protein binding was blocked by treatment with normal bovine serum albumin for 30 min. The sections were incubated overnight with anti-β_3_AR antibody (Santa Cruz Biotechnology, California, CA) at 4°C. The slides were then washed 3 times with PBS and treated with secondary antibodies for 30 min at room temperature. After washing 3 times, the slides were exposed to an ABC horseradish peroxidase (HRP) reagent (Vector Laboratories, Burlingame, CA) in PBS for 30 min. The GFP signal was developed with Peroxidase Substrate Kit AEC (Vector Laboratories), and finally the slides were mounted with water soluble mounting medium. The stained sections were visualized with an Eclipse E400 microscope (Nikon, Tokyo, Japan) attached to a high-resolution digital camera DXM 1200F (Nikon). Images were captured with ACT-1 software (Nikon).

### Quantitative Analysis of Pulmonary Arteries

Quantitative image analysis of immunohistochemical stained sections with anti-β_3_AR antibody was performed with Image Pro-Plus ver. 4.1 software as described previously [2, 25]. The red stain was selected semi-automatically. Optical density and area of the red stain were obtained. Quantification of the expression level of the protein was estimated as expression level score (ELS): ELS = (mean optical density of positively stained area–mean optical density of background area) x percent area of positively stained.

### Immunofluorescence Microscopy of Lung Sections

After deparaffinization, the lung sections were soaked in the citrate buffer and heated with microwave for 5 min. Blocking was performed with bovine serum albumin for 30 min. The sections were exposed to primary antibody overnight followed by appropriate secondary antibody for 60 min. The staining was imaged with fluorescence microscopy BIOREVO BZ-9000 (Keyence). Using primary antibodies were anti-ED1 (CD68) antibody (AbD Serotec, Oxford, UK) and anti-β_3_AR antibody (Santa Cruz Biotechnology).

### Bronchioalveolar Lavage

After 6 weeks of IH exposure, N and IH rats were sacrificed by single i.p. injection of pentobarbital and subsequent exsanguination via the abdominal aorta. The trachea was cannulated and bronchoalveolar lavage (BAL) was performed in situ by infusing the lungs with 5 mL aliquots of PBS. The BAL fluid (BALF) was drained passively by gravity and the procedure was repeated four times, giving a total BALF volume of 20 mL.

### Positive Control for Pro-inflammatory Macrophage

Pulmonary macrophages obtained from LPS administered rats were used as positive controls for pro-inflammatory macrophages. Rats were sedated by inhalation of 3% isoflurane. BALF was obtained for gathering pulmonary macrophages 24 hours after i.p. administration of LPS (10 mg/kg), after sacrificing the rats by bleeding from the abdominal aorta.

### Immunocytochemistry of Pulmonary Macrophages

BALF obtained from LPS-administered rat, N rats, and IH rats was centrifuged at 500 g for 10 min (Kubota 1720, Tokyo, Japan). Two mL of saline was added to the precipitate of BALF and mixed softly. One hundred μL of each solution was dropped onto the slides and dried overnight. The slides were fixed with cold 50% acetone in methanol for 10 min and washed three times with PBS for 5 min. They were then incubated with 1% Triton X-100 for 10 min. After being washed thrice with PBS for 5 min, the slides were blocked with bovine serum albumin for 30 min and incubated with primary antibodies overnight at 4°C. The slides were washed three times with PBS for 5 min. After incubation with fluorescent conjugated secondary antibodies, images were captured with a fluorescence microscopy BIOREVO BZ-9000 (Keyence). The primary antibodies used were anti-ED1(CD68) antibody (AbD Serotec), anti-iNOS antibody (Thermo Fisher Scientific, Waltham, MA), anti-eNOS antibody (Enzo Life Sciences, Farmingdale, NY), anti-nNOS antibody (Enzo Life Sciences), anti-CD11c antibody (AbD Serotec), anti-IL-6 antibody (R&D Systems, Minneapolis, MN), and anti-β_3_AR antibody (Santa Cruz Biotechnology).

### Positive Cell Counting in Immunofluorescent Images

For assessment of macrophage infiltration and β_3_AR upregulation, ED1 and β_3_AR positive cell counting was performed in the immunofluorescent images of lung sections. Ten representative images (200 x) were chosen from the left lobe of each animal. The number of stain positive cells was counted automatically by Image Pro Plus ver. 4.1 (Media Cybernetics).

### Nitrite Measurement of Macrophage Cultures

BALF obtained from N and IH rats were centrifuged at 500 g for 10 min (Kubota 1720, Tokyo, Japan). The pellet was resuspended in phenol red free RPMI 1640 medium (Gibco Laboratories, Grand Island, NY) with 1% streptomycin at 1.4 x 10^5^ cells / mL. The cells were plated at 4.2 x 10^5^ macrophages per well in polystyrene tissue culture plates and allowed to adhere for 12 h at 37°C in an atmosphere of 5% CO_2_ / 95% O_2_. Then, 100 μM of CL316243 (Tocris Bioscience), 100 μM of isoproterenol (LKT Laboratories, Minneapolis, MN), and 100 μM of CL316243 + 50 μM of L-NIL (Cayman Chemical) were administered. After incubation for 30 h at 37°C, the media were ultrafiltered at 7000 g x 20 min with ultracentrifugal filter units for 10 kD molecules (EMD Millipore, Billerica, MA). NO levels in the cell-free supernatant were determined by analysis of its relative stable metabolite nitrite using the Griess reaction with a fluorometric NO_2_/NO_3_ Assay Kit-FX (Dojindo Laboratories, Tokyo, Japan).

### Western blot analyses

Frozen lung tissue 0.1 g was homogenized with 1 mL of ice-cold RIPA buffer containing 0.1% SDS, 0.5% DOC, 1% NP-40, 150 mM NaCl, 50 mM Tris-Cl pH 7.4, 50 mM NaF, 1 mM Na_3_VO_4_ and Complete Protease Inhibitor Cocktail (Roche Diagnostics, Mannheim, Germany). To remove debris, the homogenate was centrifuged at 1500 g for 5 min, and supernatant was used for analysis, and the rest was frozen at -80°C. For direct detection of protein expression in macrophage, BALF obtained from N and IH-rats was centrifuged at 500 g for 5 min and 1 mL of RIPA buffer was added to the pellet. The macrophage suspension was sonicated sufficiently and centrifuged at 1000 g for 5 min to remove debris. The homogenized samples of lung and macrophage were heated at 95°C for 5 min, and 3 x Laemmli buffer containing 9% mercaptoethanol added. The supernatant was used for analysis and the rest was stored at -80°C. The protein concentration of homogenates was determined by the Bradford assay. The homogenate was subjected to SDS-PAGE on a 4–20% gradient precast gel, and separated protein was then transferred to polyvinylidene fluoride (PVDF) membranes using a transfer system Trans Blot Turbo (BioRad, Tokyo, Japan). Nonspecific antibody binding was blocked using 3% skim milk in TBS-T 0.1%, and the membranes were incubated with primary antibodies. The signals were detected by a luminescent image analyzer Image Quant Las 4000 mini (GE Healthcare, Waukesha, WI) using a secondary antibody coupled to horseradish peroxidase (Promega, Madison, WI). Primary antibodies used were anti-β_3_AR (Santa Cruz Biotechnology), anti-iNOS antibody (Abcam), anti-eNOS antibody (Enzo Life Sciences, Farmingdale), anti-nNOS antibody (Enzo Life Sciences), anti-IL-6 antibody (R&D Systems), and anti-TNFα antibody (Abcam).

### RT-PCR analysis

Lung tissue samples were homogenized and used for RNA isolation using ISOGEN (Nippon Gene, Tokyo, Japan). The purified RNA was then reverse transcribed using TaqMan Reverse Transcription Reagents (Applied Biosystems Japan, Tokyo, Japan). Expression levels of mRNA of β_3_AR were assayed quantitatively by real-time RT-PCR using TaqMan Gene Expression Assays (Applied Biosystems Japan). Quantitative mRNA expression data were acquired and analyzed by 7000 Sequence Detection System (Applied Biosystems Japan).

### Statistical analysis

All statistical analyses were conducted using GraphPad Prism6 (GraphPad Software, San Diego, CA). The results of relative expression of β_3_AR mRNA and % change in internal diameter in SR angiograms are presented as mean ± standard error of the mean (S.E.M.) and the data analysis was performed using Student’s *t*-test (unpaired) or two-way ANOVA with Sidak’s multiple comparison tests. All other data are presented as mean ± standard deviation (S.D.) and the data analyses were performed using Student’s *t*-test (unpaired) or one-way ANOVA with Tukey’s multiple comparison tests. A *P* value of < 0.05 was predetermined as the level of significance for all statistical analysis.

## Results

### IH induces macrophage accumulation and upregulated β_3_AR expression in the lungs

At first, to confirm the activation of the sympathoadrenal system in IH, the rats’ urinary concentrations of dopamine, adrenaline, and noradrenaline were measured. Urine was collected from N and IH rats over 24 hours in normoxic conditions on the day after the last day of IH exposure. The concentrations of these catecholamines were significantly higher in IH rats than in N rats (S1 Fig).

**Fig 1:**
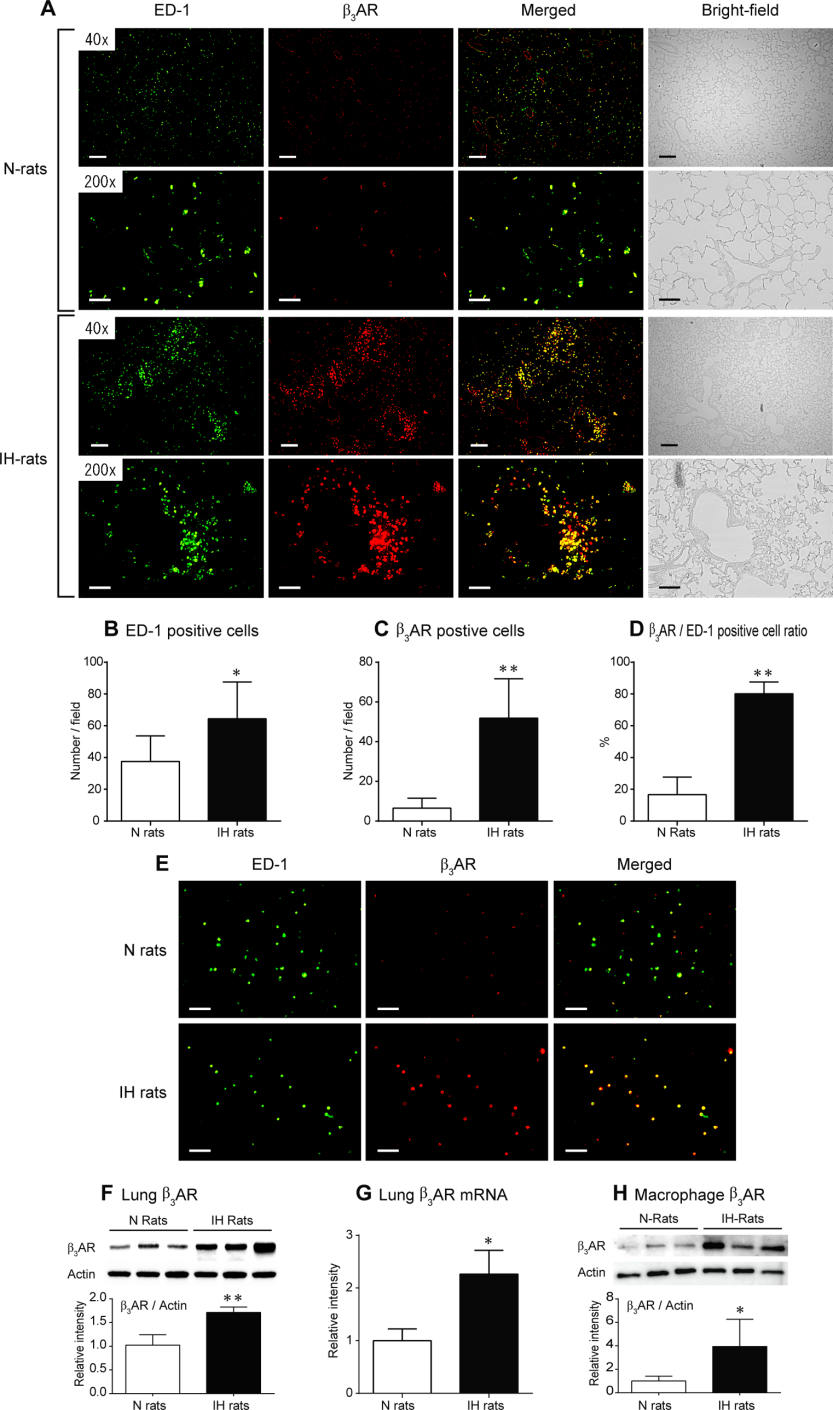
IH causes the accumulation of macrophages and upregulates β_3_AR expression in the lungs. (A) Representative bright-field images of lung sections from the N and IH rats and images of immunofluorescent staining of such sections with anti-ED-1 antibody, anti-β_**3**_AR antibody, or both (merged images). Calibration bar = 200 μm for 40 x, 50 μm for 200 x. (B, C) The numbers of ED-1- and β_**3**_AR-positive cells per field (200 x) were counted using Image Pro Plus ver. 4.1 (n = 6 each, mean ± S.D.) (D) Ratio of the percentage of β_**3**_AR-positive cells to the percentage of ED-1-positive cells (n = 6 each, mean ± S.D.) (E) Representative images of double immunocytochemical staining using anti-ED-1 and β_**3**_AR antibody in BALF-derived macrophages. Calibration bar = 50 μm. (F) Western blot analysis of β_**3**_AR in lung homogenate solutions from the N and IH rats (n = 6 each, mean ± S.D.) (G) The expression level of β_**3**_AR mRNA in lung tissue samples from the N and IH rats (n = 6 each, mean ± S.E.M.) (H) Western blot analysis of β_**3**_AR in BALF-derived pulmonary macrophages obtained after 6 weeks of IH or normoxic exposure (n = 5 each, mean ± S.D.) *Significant difference between the N and IH rats (*P<0.05, **P<0.01).

Immunofluorescent staining of pulmonary tissue performed after 6 weeks of IH exposure demonstrated that the number of pulmonary macrophages was significantly increased and the positive ratio of β_3_AR-expressing cells was high (Fig 1A–1D). The number of macrophages in the alveolar space was increased (S2 Fig). Immunocytochemistry showed that β_3_AR was strongly expressed in BALF-derived alveolar macrophages from IH rats (Fig 1E). In IH rats, macrophages accumulated around the small pulmonary arteries and these perivascular macrophages also expressed β_3_AR (Fig 1A, S3 Fig). Western blotting and RT-PCR showed that the β_3_AR was expressed in both the lung tissue and BALF-derived alveolar macrophages of N rats (Fig 1F–1H), and immunohistochemistry demonstrated that the β_3_AR was expressed on the endothelium of the pulmonary arteries (S4A Fig). IH significantly increased the protein and mRNA expression levels of β_3_AR in the lung tissues (Fig 1F and 1G). The β_3_AR protein expression was also elevated in the BALF-derived alveolar macrophages (Fig 1H). In contrast, IH decreased the expression level of β_3_AR in the pulmonary arterial endothelium of vessels with diameters ranging from 50 to 150 μm (S4B Fig). These results indicate that β_3_AR expression was upregulated in macrophages but not in the pulmonary arteries in IH rats.

To identify the origin of accumulated macrophages in the lungs of IH, intravenous administration of fluorescent liposomes was performed during IH experiments. The results of this study demonstrate that the increase in the number of pulmonary macrophages induced by IH stems from the migration of circulating monocytes into the lungs (S5A Fig). As a positive control, the liver was used for observation of fluorescent liposome engulfed monocytes. Interestingly, IH-induced accumulation of macrophages was also observed in the liver (S5B Fig).

### Pulmonary macrophages in IH rats expressed pro-inflammatory markers including iNOS

**Fig 2:**
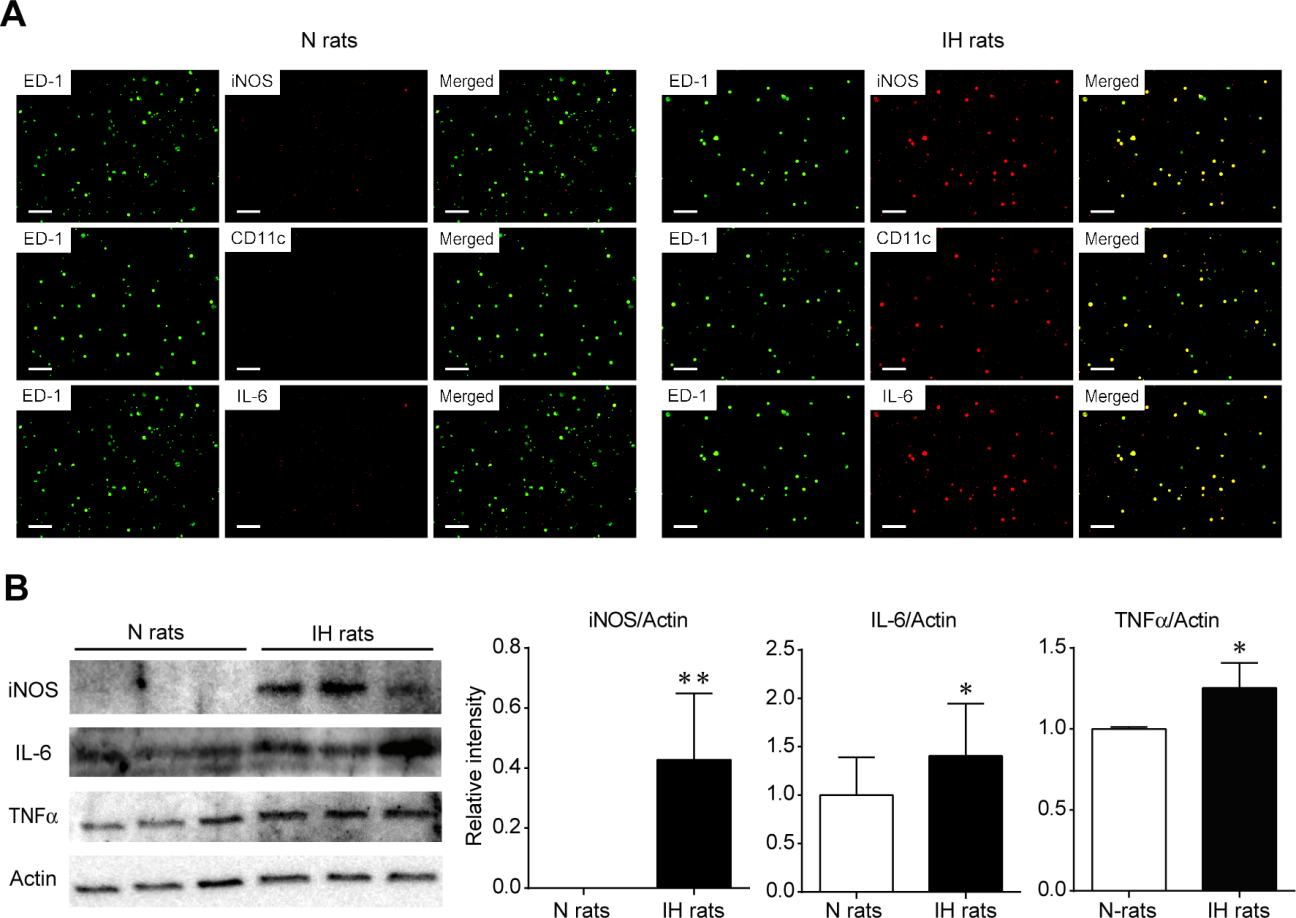
Pulmonary macrophages in IH rats expressed pro-inflammatory markers including iNOS. The pulmonary macrophages were obtained from BALF after 6 weeks of IH or normoxic exposure. (A) Immunocytochemical staining of pro-inflammatory markers iNOS, CD11c, and IL-6 was performed. (B) Western blot analysis of iNOS, IL-6, and TNFα in BALF-derived macrophages (n = 5 each, mean ± S.D.). *Significant difference between the N and IH rats (*P<0.05).

To characterize the phenotype of pulmonary macrophages in the lungs of IH, immunocytochemical staining and western blotting were performed using iNOS, CD11c, and IL-6. LPS administered rats were used for a positive control of inflammatory macrophages (S6 Fig). Pro-inflammatory markers such as iNOS, CD11c, and IL-6 were detected in IH rat macrophages, but not those of N rats (Fig 2A). Western blotting demonstrated that the protein expression levels of pro-inflammatory markers; i.e., iNOS, IL-6, and TNFα were significantly upregulated in IH-induced macrophages (Fig 2B). These results indicated that the IH stimulation promoted differentiation of the pulmonary macrophages into a pro-inflammatory type.

### Pulmonary macrophages release NO via the β_3_AR/iNOS pathway in IH rats

**Fig 3:**
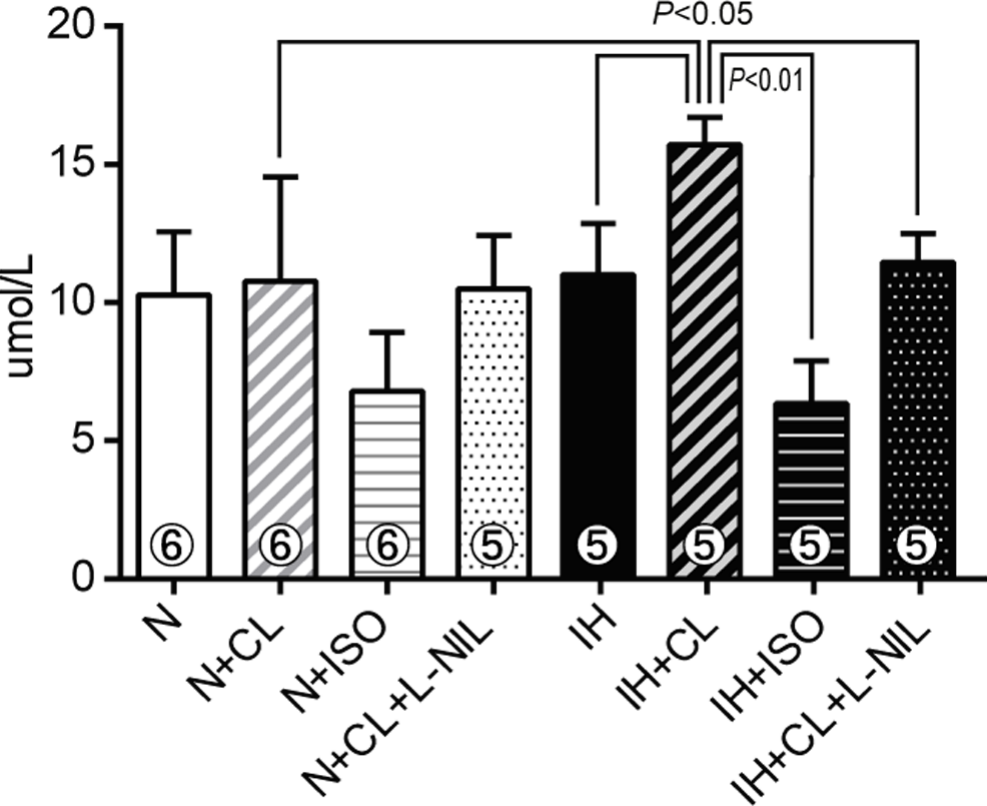
Pulmonary M1 macrophages release NO after activation of the β_3_AR/iNOS pathway in IH rats. Nitrite synthesis from BALF-derived pulmonary macrophages that had been treated with or without CL316243 (CL), isoproterenol (ISO), and CL+L-NIL. A quantitative analysis of nitrite secretion was performed 30 hours after the addition of the drugs using the Griess reaction. The number of rats in each group is shown within each column. Data are presented as mean ± S.D. values.

To assess the NO synthesis ability of pulmonary macrophages, BALF-derived macrophages were used for *in vitro* experiments. In groups without drug administration, the total amount of the macrophage-derived nitrite (chemically stable metabolite of NO) was not different between N and IH rats. In the pulmonary macrophages obtained from IH rats, but not N rats, the administration of the β_3_-agonist CL316243 enhanced the secretion of nitrite, which is indicative of elevated NO synthesis and release (Fig 3). The increase in nitrite synthesis induced by CL316243 was prevented by the simultaneous administration of the iNOS blocker L-NIL. In contrast, the non-selective β_1_ and β_2_-agonist isoproterenol decreased nitrite synthesis in both N and IH rats. These results suggest that NO secretion was facilitated in the IH-derived pro-inflammatory macrophages by the activation of β_3_AR/iNOS signaling, but not by β_1_ or β_2_AR activation.

### HPV is markedly attenuated in IH rats

**Fig 4:**
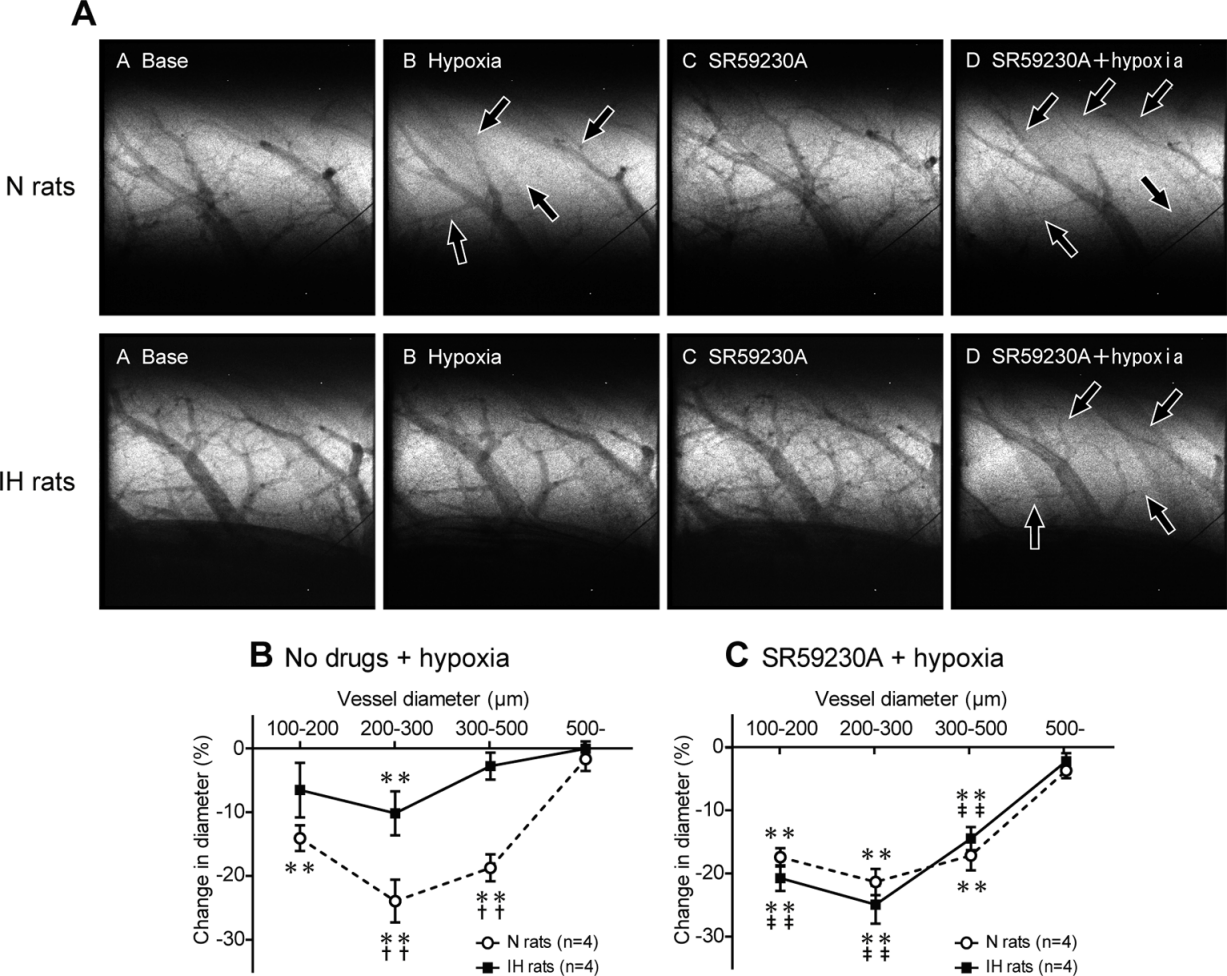
Blockade of β_3_AR completely restores attenuated HPV in IH rats. (A) Representative microangiographic images showing the branching pattern of the small pulmonary arteries during normoxia and in response to hypoxia with or without SR59230A (selective β_**3**_-blocker). Images were recorded after 5 min exposure to A: normoxia, B: hypoxia (10% O_**2**_), C: SR59230A + normoxia, and D: SR59230A + hypoxia in the same lung field. The black arrows point to branches of the pulmonary arteries that constricted in response to acute hypoxia. The tungsten wire in the lower right corner of each image is a reference wire with a diameter of 50 μm. (B, C) Relationship between vessel size and the extent of the pulmonary vasoconstriction (% decrease in vessel diameter) induced in response to acute hypoxia in the N and IH rats treated with or without SR59230A. The data are presented as mean ± S.E.M. values. *Significant reduction in vessel diameter compared with the normoxic conditions (**P<0.01). ^†^Significant difference between the N and IH rats (^††^ *P*<0.01). ^‡^Significant difference compared with the no drug conditions. (^‡‡^ *P*<0.01).

The degree of HPV was estimated using synchrotron radiation microangiography. In N rats, acute hypoxic exposure (10% O_2_) induced marked constriction (HPV) in the small pulmonary arteries with internal diameters (ID) of 100–500 μm, but not in those with ID of more than 500 μm (Fig 4A and 4B). The extent of the HPV (% reduction in ID caused by acute hypoxia) tended to increase as arterial diameter decreased, with the greatest degree of constriction (approximately 24%) occurring in the arteries with ID of between 200 and 300 μm. In IH rats, acute hypoxic exposure only induced significant HPV in the pulmonary vessels with ID of 200–300 μm, and the degree of HPV was approximately half of that seen in N rats (Fig 4B), indicating that the HPV induced by acute hypoxic exposure was greatly attenuated in IH rats.

### β_3_AR activation causes iNOS-dependent vasodilation and attenuates HPV in IH

**Fig 5:**
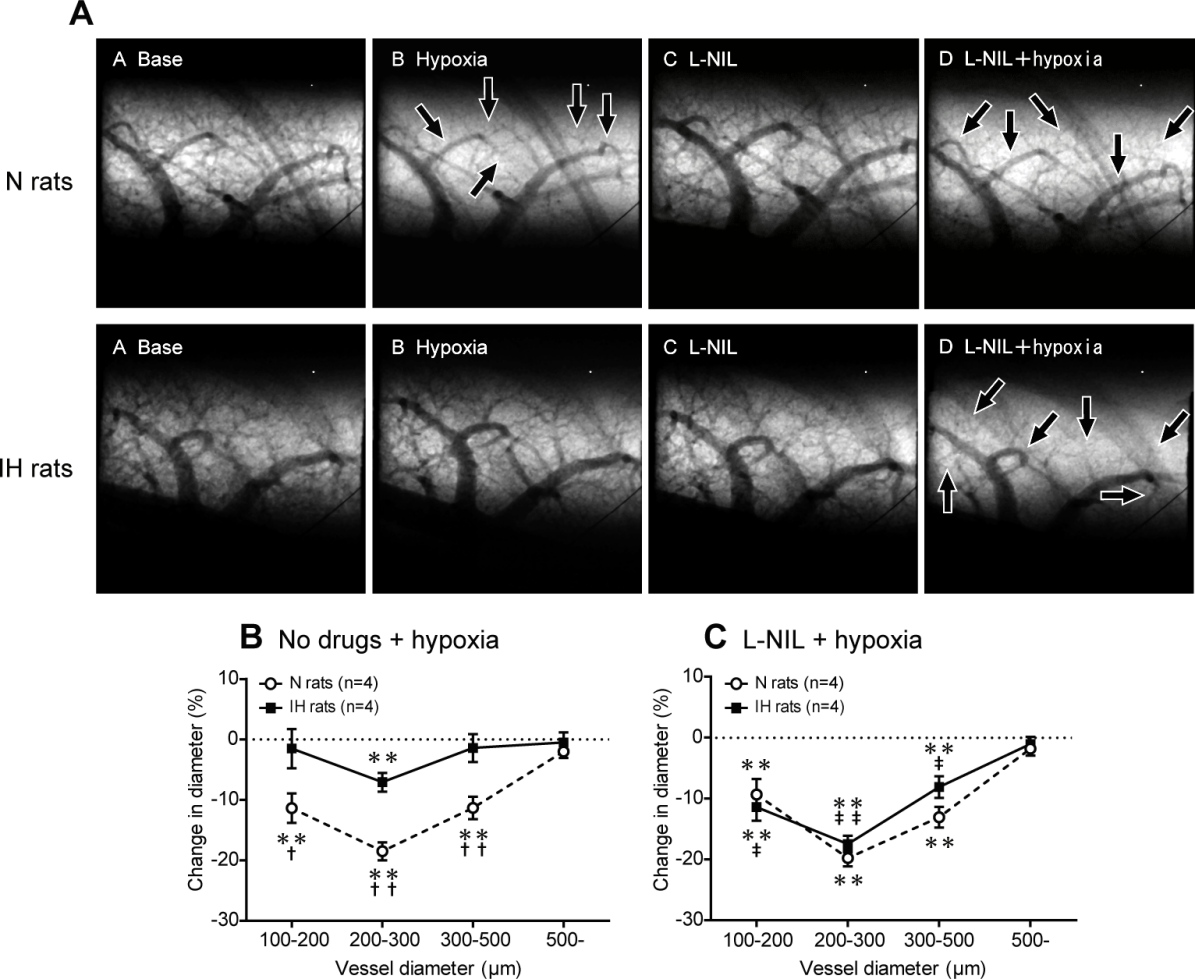
Blockade of iNOS completely restores attenuated HPV in IH rats. (A) Representative images of the branching pattern of the small pulmonary arteries at the baseline and after the administration of L-NIL (selective iNOS inhibitor). The black arrows point to constricted pulmonary arteries. (B, C) Relationship between vessel size and the extent of the pulmonary vasoconstriction induced in response to acute hypoxia with or without selective L-NIL treatment. Data are presented as mean ± S.E.M. values. *Significant change in vessel diameter compared with the baseline conditions (**P<0.01). ^†^Significant difference between the N and IH rats (^†^ *P*<0.05; ^††^ *P*<0.01). ^‡^Significant difference compared with the no drug conditions (^‡^ *P*<0.05, ^‡‡^ *P*<0.01).

In IH rats, SR59230A, a lipophilic selective β_3_-blocker, restored the attenuated HPV to almost the same level as was seen in N rats (Fig 4A and 4C). In contrast, SR59230A had no significant effect on the HPV seen in N rats. Pretreatment with the selective iNOS inhibitor L-NIL also restored the attenuated HPV in the same manner as SR59230A (Fig 5). These results suggest that IH activates both a β_3_AR-mediated and iNOS-mediated vasodilatory mechanism to attenuate HPV.

**Fig 6:**
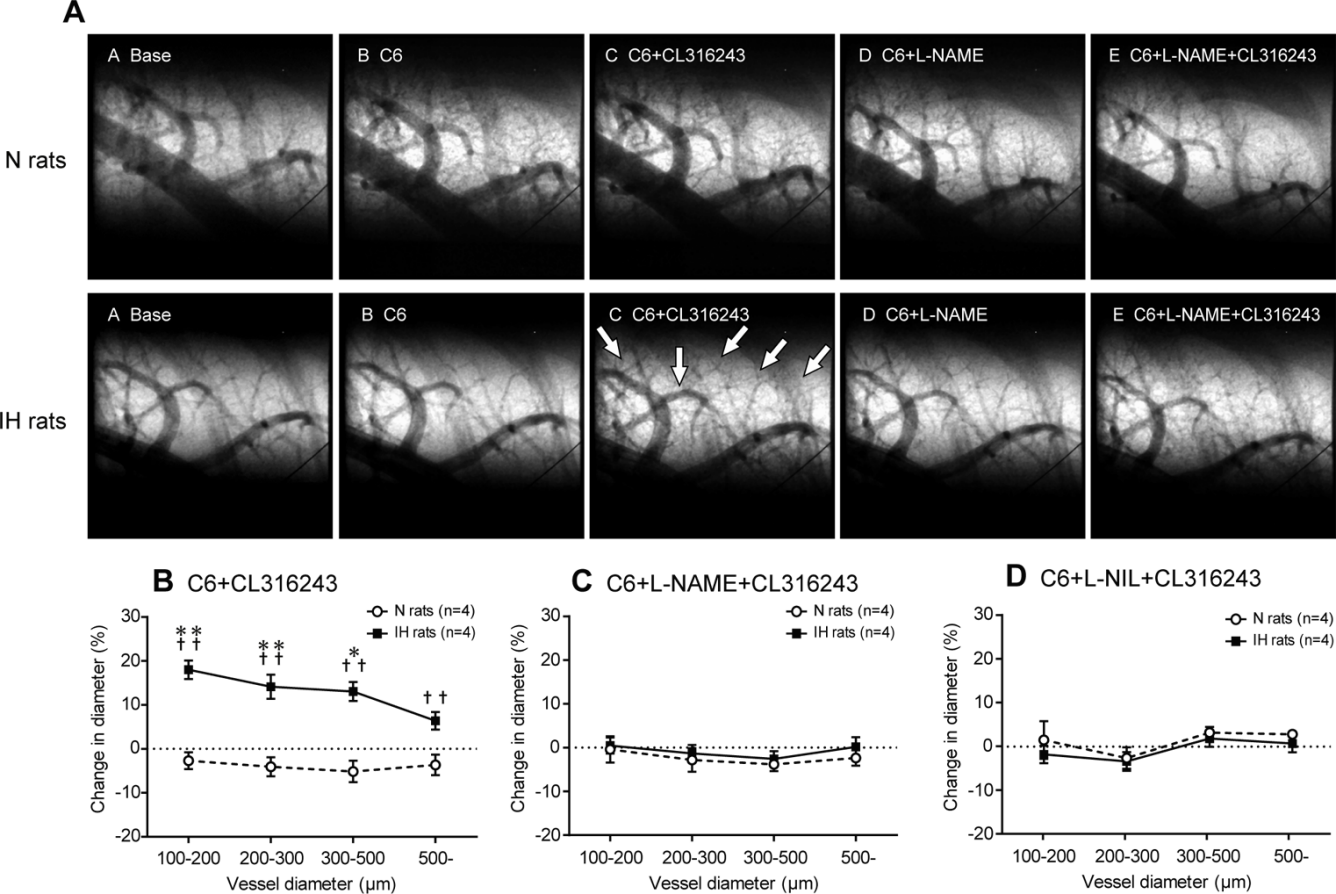
Stimulation of β_3_AR dilates pulmonary arteries via iNOS-dependent signaling in IH rats. (A) Representative microangiographic images showing the branching pattern of the small pulmonary arteries at the baseline and after the administration of each drug. The white arrows point to branches of dilated pulmonary arteries or small pulmonary arteries that were first detected after the administration of CL316243 (a selective β_**3**_-agonist). The tungsten wire in the lower right corner of each image is a reference wire that measures 50 μm in diameter. (B) Extent of the change in vessel diameter induced in response to the administration of CL316243 with pretreatment of hexamethonium bromide (C6) in the N and IH rats. Data are presented as mean ± S.E.M. values. *Significant change in vessel diameter compared with the baseline conditions (*P<0.05; **P<0.01). ^†^Significant difference between the N and IH rats (^†^ *P*<0.05; ^††^ *P*<0.01). (C, D) Percentage change in the mean diameter of small pulmonary arteries in response to the administration of CL316243 after pretreatment of with either L-NAME or L-NIL. Data are presented as mean ± S.E.M. values.

To assess whether peripheral β_3_AR, but not the β_3_AR in the central nervous system, contribute to HPV attenuation in IH, CL316243 was administered under ganglionic blockade with hexamethonium bromide. In IH rats, CL316243 induced extensive dilatation of the small pulmonary arteries, particularly in the arteries with ID of 100–500 μm (Fig 6A and 6B). In contrast, CL316243 had no significant effect in N rats. These results indicate that peripheral β_3_AR were activated to a much greater extent in IH rats than in N rats, and strongly suggest that peripheral β_3_AR contributed to the HPV attenuation observed in IH rats.

Next, we investigated whether NOS is involved in the β_3_AR-mediated pulmonary vasodilation observed in IH rats. Pretreatment with L-NAME or L-NIL completely abrogated the CL316243-mediated pulmonary vasodilation in IH rats (Fig 6A, 6C and 6D). There was no significant difference in the strength of the inhibitory effect between L-NAME and L-NIL. These results suggest that iNOS-derived NO was chiefly responsible for the β_3_AR-induced pulmonary vasodilation observed in IH rats. Taken together, these observations strongly suggest that the β_3_AR/iNOS pathway is activated in IH, resulting in the dilation of pulmonary arteries and the attenuation of HPV.

### Acute intra-tracheal administration of clodronate restores HPV in IH rats

**Fig 7:**
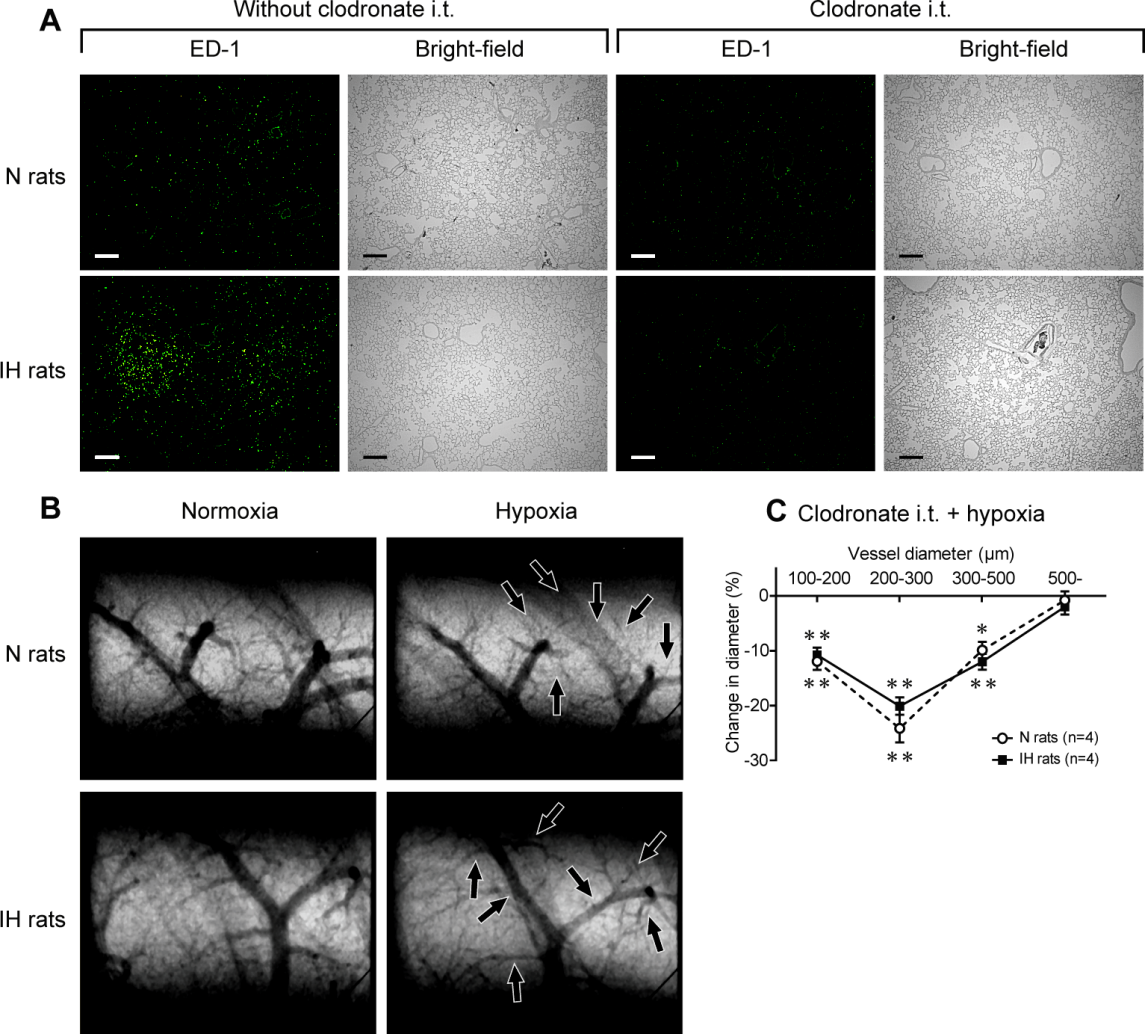
Acute intratracheal administration of clodronate restores HPV in IH rats. (A) Representative bright-field images and images of immunofluorescent staining using anti-ED-1 antibody of lung sections with or without clodronate. Clodronate (500 μg of clodronate in 100 μL of saline) was injected intratracheally just after the end of the 6-week IH/normoxia exposure period. Calibration bar = 200 μm. (B) Representative microangiographic images of the small pulmonary arteries in the N and IH rats obtained 3 days after the i.t. administration of clodronate. The black arrows point to branches that underwent vasoconstriction. (C) Relationship between vessel size and the extent of the pulmonary vasoconstriction induced in response to acute hypoxia. Data are presented as mean ± S.E.M. values. *Significant change in vessel diameter compared with the baseline conditions (**P<0.01).

To investigate whether the macrophages that accumulated in the lungs of the IH rats contributed to the observed HPV attenuation, HPV was evaluated in pulmonary macrophage depleted rats using microangiography. Clodronate was administered into the lungs of IH rats via the trachea immediately after the 6-week IH exposure period. Clodronate strongly suppressed the number of pulmonary macrophages in IH rats to the control level (Fig 7A) and enhanced the attenuated HPV to almost the same level as that seen in N rats (Fig 7B and 7C). These results indicate that the intra-alveolar macrophages that accumulate in the lungs in IH contribute to the attenuation of HPV.

## Discussion

The present study demonstrated that 1) chronic IH increases pro-inflammatory macrophages with upregulation of β_3_AR and iNOS in the lungs, 2) IH-derived activation of β_3_AR/iNOS signaling promotes NO secretion from pulmonary macrophages, and 3) that the pulmonary macrophages attenuate HPV via the β_3_AR/iNOS signaling pathway in IH rats.

We first confirmed that activation of sympathoadrenal system persists even after chronic IH exposure. Because the 24-hour collection of urine was performed while the rats were maintained in a normoxic atmosphere on the day after the last day of IH exposure, the results of urinary concentrations indicate that the sympathoadrenal system is persistently activated in IH rats even after their release from chronic IH. These results are consistent with the findings of previous reports [8, 11, 26].

The present angiographic data showed that in N rats, HPV occurs in the small pulmonary arteries of 100–500 μm ID with a maximum constriction in the 200–300 μm range. This ID-dependent vasoconstriction pattern is consistent with our previous reports [11, 12, 27]. HPV was significantly attenuated in the arteries with ID ranging between 100–500 μm and significant HPV was observed only in the arteries of 200–300 μm ID following IH-induced sympathoadrenal activation. This result is also consistent with our previous report [11, 12]. The present study has revealed that this attenuation of HPV is abrogated by β_3_AR blockade. Further, after chemical blocking of the sympathetic nerve with a ganglion blocker, stimulation of β_3_AR induced significant dilation in the small pulmonary arteries in IH rats, but not in N rats. Collectively, these results suggest that in IH, peripheral β_3_AR which are distributed within the lungs are activated by increased sympathoadrenal activity, causing the decrease in pulmonary vascular tone and attenuation of HPV.

In N rats, β_3_AR was observed in the endothelium of the small pulmonary arteries; however, β_3_-agonist had no significant vasodilatory effect on these vessels. This is consistent with the findings of previous studies in which β_3_AR had no [28, 29] or only weak vasodilatory capacity in normoxic pulmonary vessels [30, 31]. In addition, we showed that the β_3_AR expression in the small pulmonary arteries is decreased in IH rats. Collectively, pulmonary vascular β_3_AR are likely to play a minimal role in controlling vascular tone in IH. In contrast, we showed that the β3AR expression on the alveolar and the perivascular macrophages is significantly elevated in IH rats. Moreover, the depletion of intra-alveolar macrophages restored the normal level of HPV in IH rats. These results suggest that the β3AR expressed on the more abundant ‘alveolar’ macrophages contribute to attenuation of HPV in IH rats. In the present study, it was not elucidated whether the ‘perivascular’ macrophages contributed to attenuation of HPV in the same manner as alveolar macrophages. The high expression of the β3AR in the perivascular macrophages implicates their contribution to HPV modification, although future research is essential to resolve this question.

Several subsets of macrophages with distinct functions have been described. M1 macrophages promote inflammation to defend the host from a variety of foreign bodies. M2 macrophages have anti-inflammatory functions and regulate wound healing [32]. Previously, accumulation of macrophages in the lungs [33] and around the pulmonary arteries [34] has been reported in chronic hypoxic experiments. Frid et al. showed that monocyte-derived macrophage infiltration/accumulation in the adventitia of pulmonary arteries was observed after 4 weeks of chronic hypobaric hypoxia (380 mmHg) in rats [34]. Vergadi et al. showed that accumulation of macrophages in the lungs was observed from 2 days of chronic hypoxia in the mouse, and a significant increase in the number of macrophages maintained throughout the 2 weeks of a hypoxic experiment period [33]. Importantly, these macrophages accumulating with chronic hypoxia-induction were M2 type. In our experiments, accumulation of macrophages in the lungs was observed after 6 weeks of IH exposure. The alveolar macrophages expressed pro-inflammatory proteins such as iNOS, CD11c, and IL-6, suggesting that these macrophages were M1 type. However, to elucidate the phenotype of these macrophages, more detail evaluation including characterization of their gene expression profile is needed. Taken together, the present data indicate that the IH-derived stimulation of β_3_AR promoted differentiation of the pulmonary macrophages into a pro-inflammatory type with expression of iNOS. Thus, IH and chronic hypoxia have different effects on the polarization of macrophages. Moreover, chronic intravenous administration of fluorescent liposomes demonstrated that IH induced the migration of circulating monocytes into the lungs. However, the detailed mechanisms responsible for migration of the circulating monocytes are not revealed in the present study. Since the migration ability of circulating monocytes into the local peripheral tissues depends on the expression level of chemokine/chemoreceptors [35], further study is needed to elucidate the migration mechanism of monocytes into the lungs due to chronic IH exposure.

In the present *in vitro* study, we demonstrated that β_3_AR stimulation increased NO secretion via iNOS activation in IH pulmonary macrophages, but not in N macrophages (Fig 3). In cardiomyocytes, all subtypes of NOS; i.e., eNOS, nNOS, and iNOS, were reported to be downstream of β_3_AR signaling [17, 36–39]. Most of these studies detected significant correlations between β_3_AR and eNOS expression. To the best of our knowledge, there has been only one report about the relationship between β_3_AR and iNOS [17]. In the present study, eNOS was detected in the alveolar macrophages (S7A Fig); however, the protein expression level of eNOS did not differ between IH and N rats (S7B Fig). eNOS expressed on unactivated macrophages is known to secrete a small amount of NO continuously [40]. Therefore, in the present *in vitro* study, the baseline level of nitrite secretion from the macrophages in the absence of drug treatment probably originated from eNOS in both N and IH rats. On the other hand, iNOS expressed in IH-derived macrophages is suggested as the most dominant physiologically relevant source of NO from the macrophages [41]. Additionally, this previous report revealed that macrophage-derived NO directly dilates femoral arterial rings in *ex vivo* experiments [42]. Therefore, considering these findings and our results for angiography (Figs 4–7), it is strongly indicated that pro-inflammatory macrophages attenuate the HPV via β_3_AR/iNOS pathway-derived NO secretion.

In contrast to the results of selective β_3_AR stimulation, isoproterenol inhibited NO secretion in both N and IH macrophages. This result is consistent with a previous report in which catecholamines inhibited the macrophage-mediated production of NO through β_1_ and β_2_AR *in vitro* [43, 44]. Recently, it has been revealed that phosphorylation sites for protein kinase A and βAR kinase are found in the β_1_ and β_2_AR, whereas the β_3_AR lacks these sites. Thus, the β_1_ and β_2_AR undergo functional desensitization after long-term exposure to hypercatecholemia. In contrast, sustained stimulation of the β_3_AR does not modify its functional effects [45–47]. In the present study, function of the β_1_ and β_2_AR on the pulmonary macrophages was not disrupted after 6 weeks of IH exposure, however, there is a possibility that much longer exposure of IH than 6 weeks decreases the inhibitory effects of NO production from the macrophages. Accordingly, we suggest that β_3_AR signaling probably plays a pivotal role in controlling the pulmonary circulation in the pathogenesis accompanying prolonged sympathoadrenergic activation such as chronic IH.

In our previous study, the β_2_AR dependent activation of PI3kinase/Akt/eNOS signaling in the pulmonary arteries attenuated the HPV, leading to the prevention of the progression of pulmonary arterial hypertension (PAH) [12]. Interestingly, blockade of β_2_AR and β_3_AR exacerbated HPV in IH rats to the same degree as that in control rats. These findings suggest that both β_2_AR and β_3_AR have critical contribution to attenuate HPV in IH. Taking these findings into consideration, the β_3_AR/iNOS pathway in pro-inflammatory macrophages presumably behave in the same manner as the β_2_AR/eNOS pathway in pulmonary arteries to prevent PAH progression in IH. To confirm this hypothesis, further research is required.

In SAS patients, PaCO_2_ is increased due to obstruction of the upper airway during sleeping periods [9]. The effect of PaCO_2_ on the pulmonary vascular function in SAS has not been elucidated, however it has been reported that supplementation of CO_2_ significantly attenuates HPV in rat [48, 49]. Therefore, there is a possibility that hypercapnia attenuates IH-induced HPV. To elucidate an effect of blood CO_2_ on HPV, additional experiments utilising simultaneous exposure of intermittent hypoxia and hypercapnia are needed. The present study is important with respect to focus on the effect of intermittent hypoxia to pulmonary hemodynamics, which is one of the major factors in the pathophysiology in SAS.

In summary, we demonstrate that pro-inflammatory pulmonary macrophages attenuate HPV via the activation of β_3_AR/iNOS signaling in IH rats. The relationship between IH-induced sympathoadrenal activation and pulmonary circulation is not fully elucidated, although, this study highlights the pivotal role of sympathoadrenal activation and pro-inflammatory macrophages in attenuating the HPV in IH. Moreover, we also highlight the importance of β_3_AR/iNOS signaling pathway in the preservation of the pulmonary circulation under prolonged IH exposure.

## Supporting Information

S1 Fig The mean urinary catecholamine concentrations of the IH rats were extremely high compared with those of the control rats. The concentrations of dopamine, adrenaline, and noradrenaline in 24-hour urine samples from N and IH rats (n = 5 each). Urine was collected over 24 hours using metabolic cages under a normoxic atmosphere on the day after the end of a 6-week period of normoxic or intermittent hypoxic exposure. The data are presented as mean ± S.D. values. *Significant difference between the N and IH rats (**P<0.01).
(TIF)

S2 Fig An increase in the number of macrophages is observed in alveolar spaces. Representative images of immunohistochemical staining with anti-β_3_AR antibody in paraffin embedded lung sections. (A) Almost all intra-alveolar cells in IH rats were strongly stained by anti-β_3_AR antibody. In contrast, these cells in N rats were not. These results suggest that the brown cells are macrophages. Nuclei were counterstained with Haematoxylin. Calibration bar = 10 μm. (B) These images show the distribution of macrophages in alveolar spaces. Calibration bar = 50 μm. (C) The number of macrophages in alveolar spaces were significantly increased in IH rats compared to these in N rats (n = 6 each, mean ± S.D.). *Significant difference between N and IH rats (**P<0.01).
(TIF)

S3 Fig Macrophages accumulated around a small pulmonary artery. Representative image of immunohistochemical staining with anti-β_3_AR antibody in a paraffin embedded lung sections. Anti-β_3_AR antibody stained macrophages were accumulated around a small pulmonary vessel in an IH-treated rat. Calibration bar = 20 μm.
(TIF)

S4 Fig Expression of β_3_AR is downregulated in pulmonary arteries. (A) Representative images of immunohistochemical staining with anti-β_3_AR antibody in small pulmonary arteries. Calibration bar = 20 μm. (B) Relative expression level of β_3_AR protein in small pulmonary arteries with the diameter range of 50 to 150 μm (n = 6 each, mean ± S.D.). Quantification of the expression level of the protein was estimated as expression level score (ELS): ELS = (mean optical density of positively stained area–mean optical density of background area) x percent area of positively stained. *Significant difference between N and IH rats (*P<0.05, **P<0.01).
(TIF)

S5 Fig IH induces the accumulation of circulating monocytes in the lung and the liver. Fluorescent liposomes were administered intermittently (once every 4 days) during the 6 weeks of experiments. (A) Circulating monocytes accumulated in the lungs of IH rats. (B) The images of liver were used for a positive control for circulating monocyte-derived macrophages. Calibration bar = 200 μm.
(TIF)

S6 Fig Immunocytochemical staining of pro-inflammatory markers in BALF-derived pulmonary macrophages obtained from LPS-administered rats. BALF-derived pulmonary macrophages obtained from LPS (10 mg/kg, i.p., 24h) treated rats were used as positive controls for pro-inflammatory macrophages. Immunocytochemical staining of iNOS, CD11c, and IL-6 was performed. Calibration bar = 50 μm.
(TIF)

S7 Fig eNOS and nNOS are not upregulated in BALF-derived macrophages in IH rats. (A) Representative images of double immunocytochemical staining with anti-ED-1, and eNOS or nNOS antibody in BALF-derived macrophages. Calibration bar = 50 μm. (B) Western blot analysis of eNOS in BALF-derived macrophages (n = 5 each, mean ± S.D.) nNOS was undetectable in both N and IH rats.
(TIF)
