# Supplementary figures and images for: Pulmonary Macrophages Attenuate Hypoxic Pulmonary Vasoconstriction via β3AR/iNOS Pathway in Rats Exposed to Chronic Intermittent Hypoxia
Source: PLoS One. 2015 Jul 1;10(7):e0131923. doi: 10.1371/journal.pone.0131923 (PMC4489089; doi:10.1371/journal.pone.0131923)

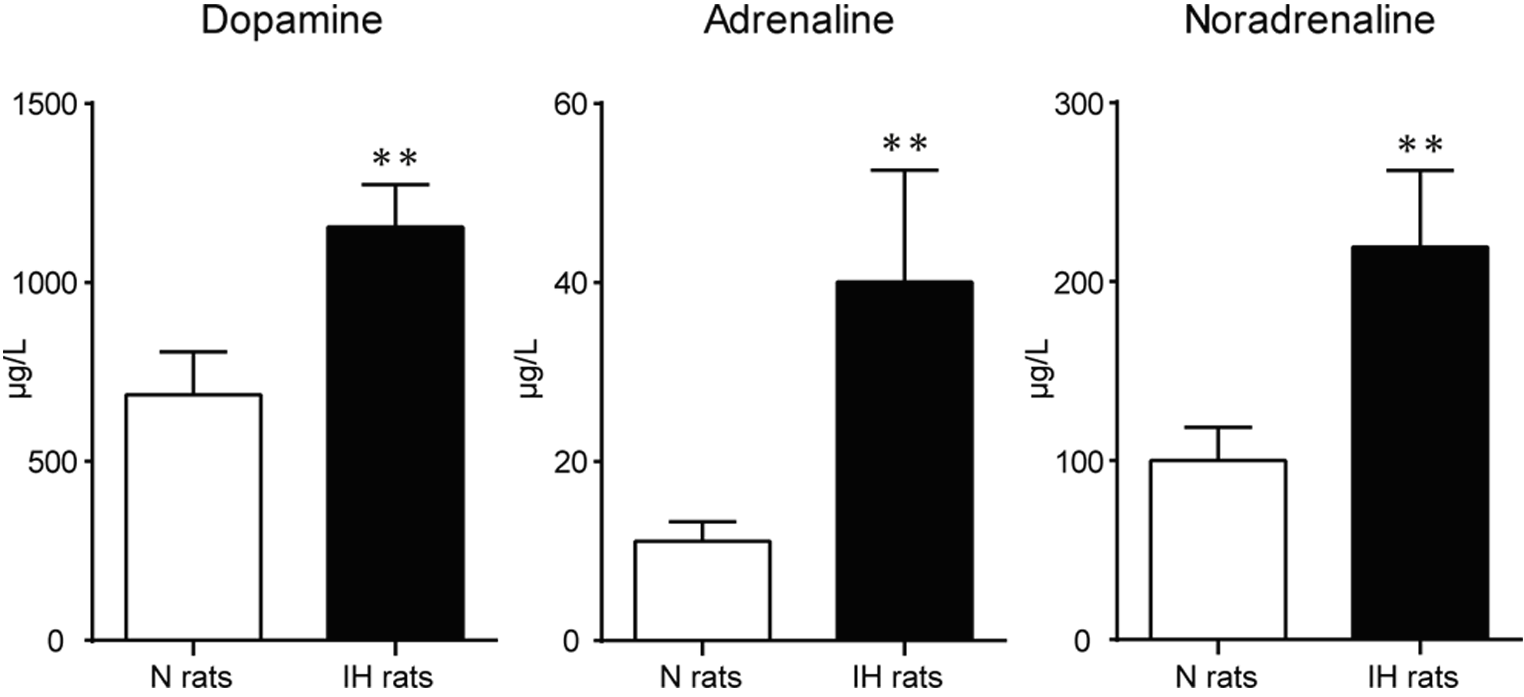

Supplement: S1 Fig — The concentrations of dopamine, adrenaline, and noradrenaline in 24-hour urine samples from N and IH rats (n = 5 each). Urine was collected over 24 hours using metabolic cages under a normoxic atmosphere on the day after the end of a 6-week period of normoxic or intermittent hypoxic exposure. The data are presented as mean ± S.D. values. *Significant difference between the N and IH rats (**P<0.01). (TIF) [file pone.0131923.s001.tif]

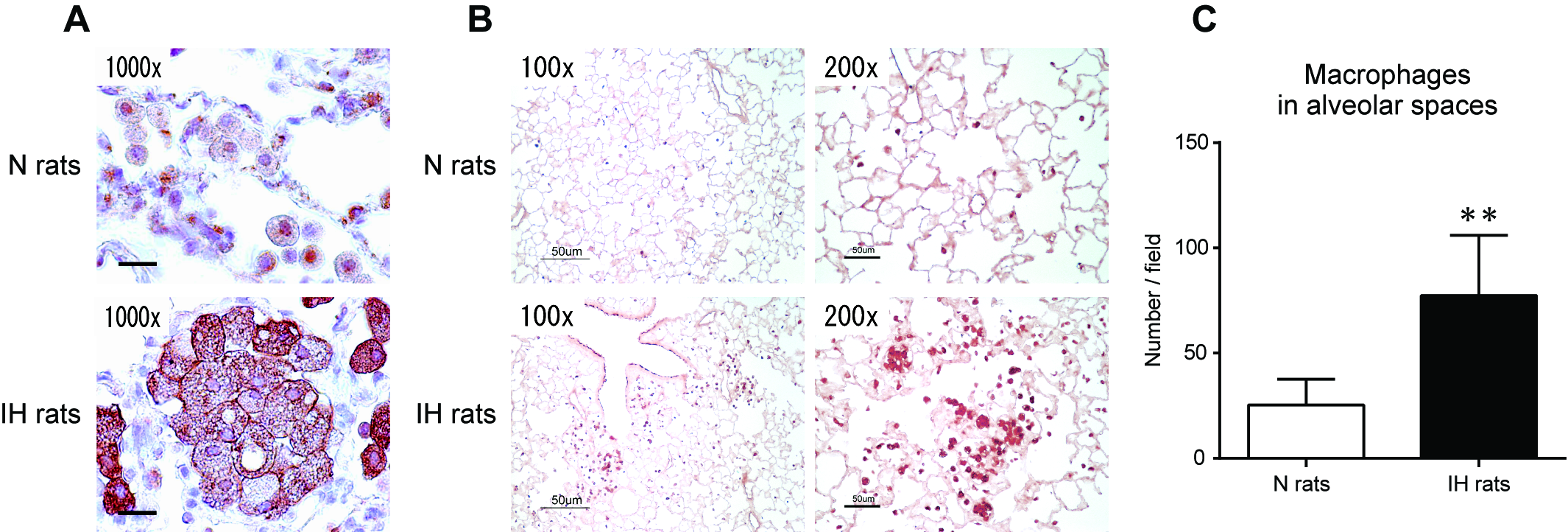

Supplement: S2 Fig — Representative images of immunohistochemical staining with anti-β3AR antibody in paraffin embedded lung sections. (A) Almost all intra-alveolar cells in IH rats were strongly stained by anti-β3AR antibody. In contrast, these cells in N rats were not. These results suggest that the brown cells are macrophages. Nuclei were counterstained with Haematoxylin. Calibration bar = 10 μm. (B) These images show the distribution of macrophages in alveolar spaces. Calibration bar = 50 μm. (C) The number of macrophages in alveolar spaces were significantly increased in IH rats compared to these in N rats (n = 6 each, mean ± S.D.). *Significant difference between N and IH rats (**P<0.01). (TIF) [file pone.0131923.s002.tif]

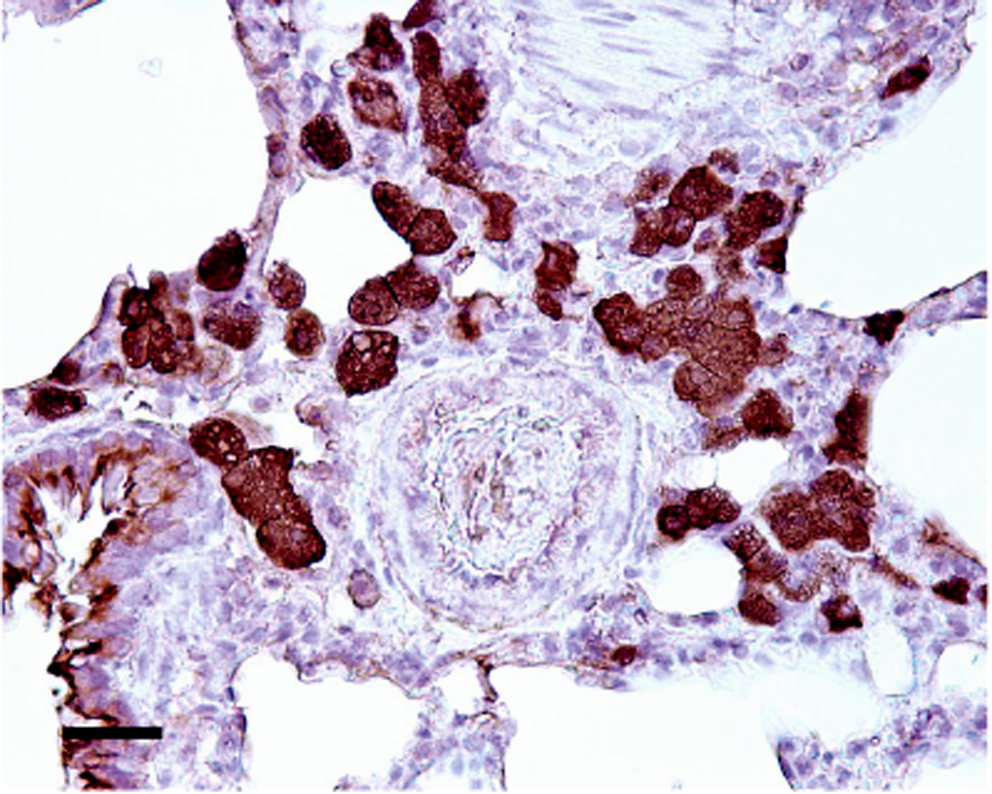

Supplement: S3 Fig — Representative image of immunohistochemical staining with anti-β3AR antibody in a paraffin embedded lung sections. Anti-β3AR antibody stained macrophages were accumulated around a small pulmonary vessel in an IH-treated rat. Calibration bar = 20 μm. (TIF) [file pone.0131923.s003.tif]

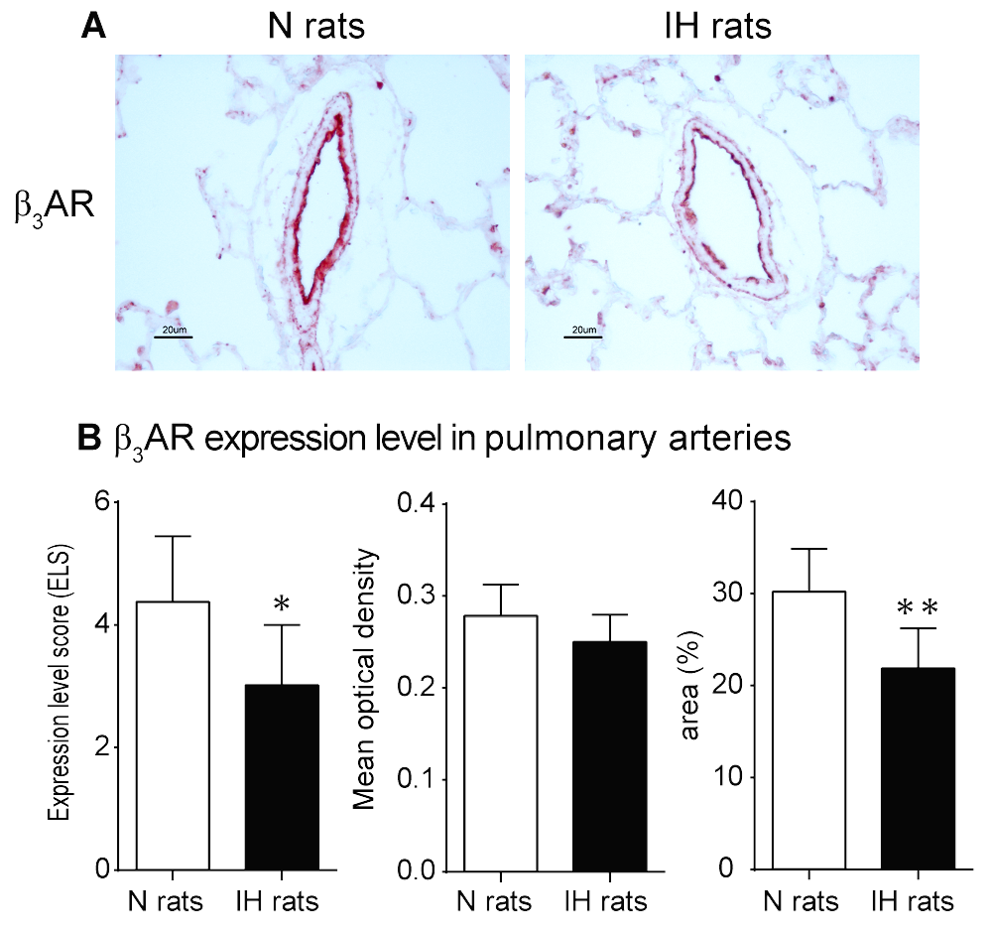

Supplement: S4 Fig — (A) Representative images of immunohistochemical staining with anti-β3AR antibody in small pulmonary arteries. Calibration bar = 20 μm. (B) Relative expression level of β3AR protein in small pulmonary arteries with the diameter range of 50 to 150 μm (n = 6 each, mean ± S.D.). Quantification of the expression level of the protein was estimated as expression level score (ELS): ELS = (mean optical density of positively stained area–mean optical density of background area) x percent area of positively stained. *Significant difference between N and IH rats (*P<0.05, **P<0.01). (TIF) [file pone.0131923.s004.tif]

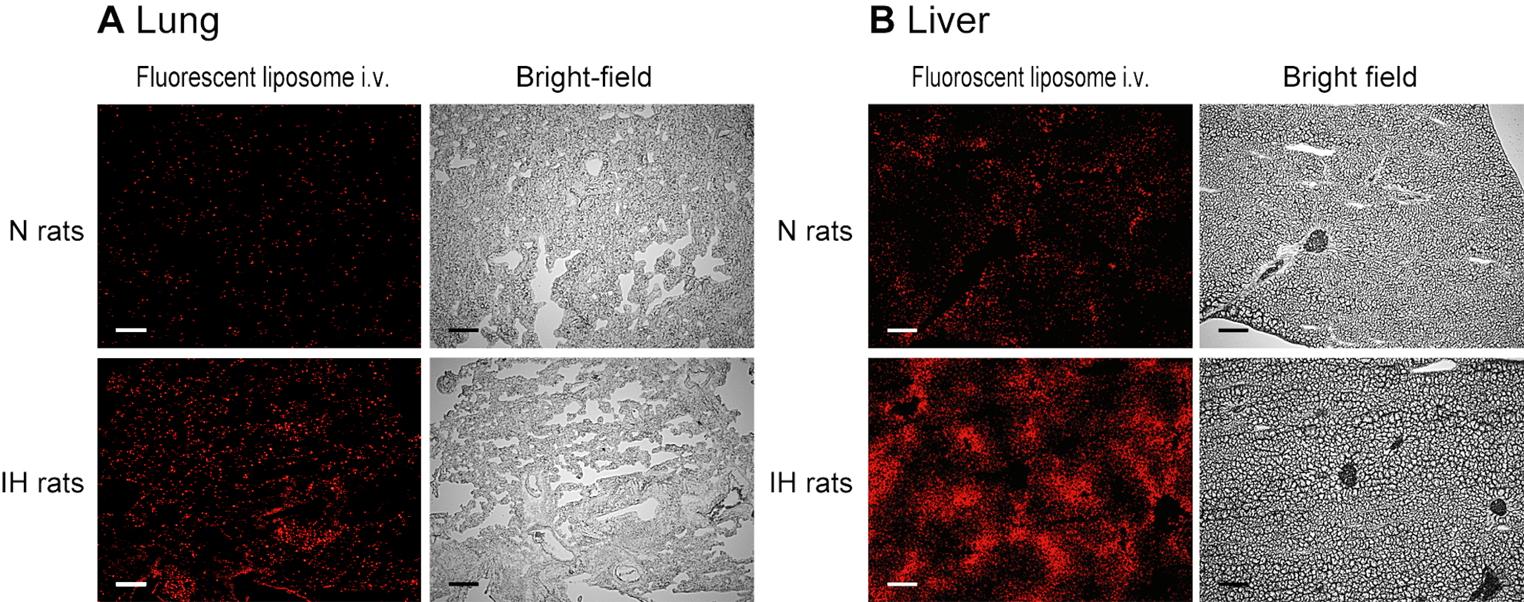

Supplement: S5 Fig — Fluorescent liposomes were administered intermittently (once every 4 days) during the 6 weeks of experiments. (A) Circulating monocytes accumulated in the lungs of IH rats. (B) The images of liver were used for a positive control for circulating monocyte-derived macrophages. Calibration bar = 200 μm. (TIF) [file pone.0131923.s005.tif]

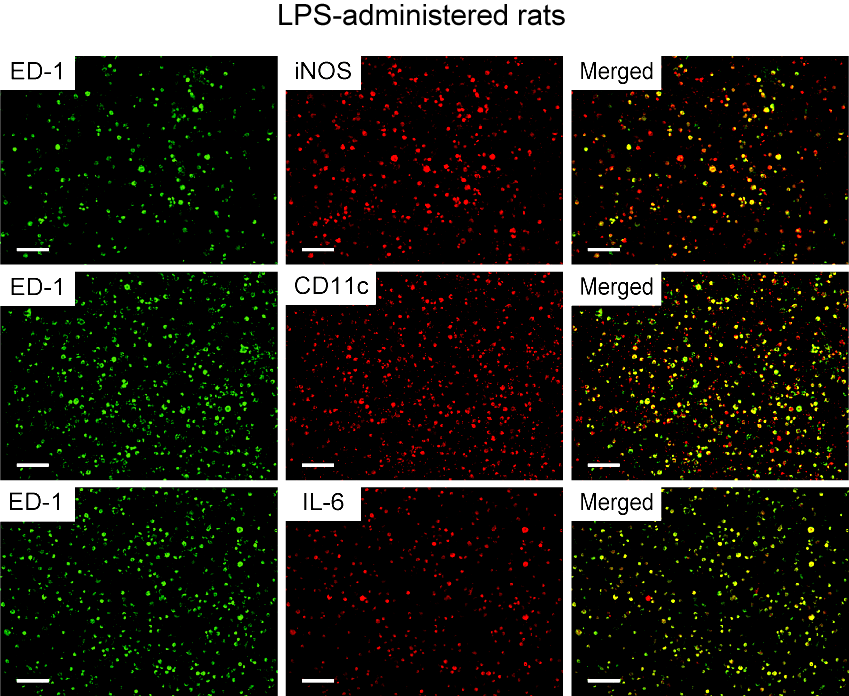

Supplement: S6 Fig — BALF-derived pulmonary macrophages obtained from LPS (10 mg/kg, i.p., 24h) treated rats were used as positive controls for pro-inflammatory macrophages. Immunocytochemical staining of iNOS, CD11c, and IL-6 was performed. Calibration bar = 50 μm. (TIF) [file pone.0131923.s006.tif]

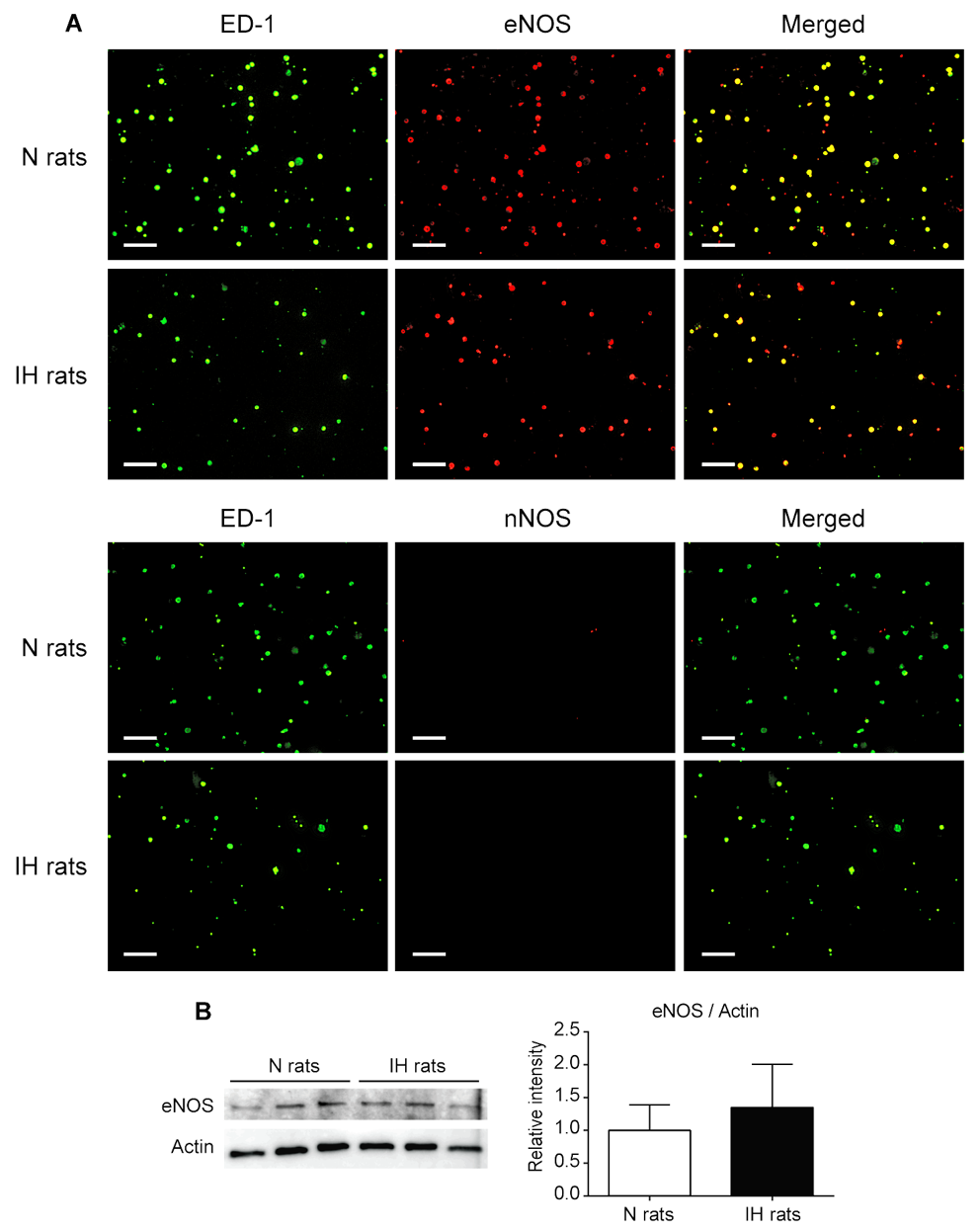

Supplement: S7 Fig — (A) Representative images of double immunocytochemical staining with anti-ED-1, and eNOS or nNOS antibody in BALF-derived macrophages. Calibration bar = 50 μm. (B) Western blot analysis of eNOS in BALF-derived macrophages (n = 5 each, mean ± S.D.) nNOS was undetectable in both N and IH rats. (TIF) [file pone.0131923.s007.tif]
